# Together we win! Narratives of couples pursuing a healthy diet and physical activity: A qualitative study

**DOI:** 10.1002/hsr2.70022

**Published:** 2024-08-28

**Authors:** Sundus Nizamani, Catherine R. Knight Agarwal, Shawn Somerset, R. A. McFarlane

**Affiliations:** ^1^ Discipline of Public Health Faculty of Health, University of Canberra Canberra Australia; ^2^ Department of Nutrition & Dietetics Faculty of Health, University of Canberra Canberra Australia

**Keywords:** adherence, couples, lifestyle intervention, metabolic syndrome, preconception, weight

## Abstract

**Background and Aims:**

Metabolic syndrome (MetS) is a major risk factor for non‐communicable diseases, including type 2 diabetes mellitus, cardiovascular disease, and cancer. The risk of MetS can be transmitted via epigenetic processes from both the mother and the father. Therefore, it is essential that both members of a couple are targeted in pre‐conception nutrition and physical activity‐based lifestyle programs. However, lifestyle interventions targeting both members of a couple are scarce in the literature. This study, therefore, aimed to explore the barriers and facilitators of a couples‐based lifestyle intervention.

**Methods:**

Nulliparous couples who had an interest in having children in the future were recruited to the study and each member interviewed separately to gain insight into to gain insight into designing future couples‐based lifestyle interventions. Interviews were conducted between June and October 2021. Reflexive thematic analysis (RTA) was applied to conduct and analyse semi‐structured, in‐depth interviews with nulliparous couples who had an interest in having children in the future.

**Results:**

Four major themes were identified in nine couples aged 25–34 years (here referred to as “millennials”): Millennials are committed to preparation for their future offspring; millennials know features of a good program likely to have high adherence and long‐term behavior change; millennials acknowledge the importance of couples‐based programs; and millennials appreciate that future global emergencies may require lifestyle modifications.

**Conclusion:**

This study found that millennial couples showed a strong intention to create optimal emotional, financial, and health conditions for their children. They supported couples‐based approach (CBA) lifestyle interventions to mitigate potential epigenetic risks. Couples believed that participating together in these programs would enhance adherence to healthy habits, promoting long‐term well‐being. The findings advocate for exploring and testing CBA interventions that target both partners, as joint participation not only aids in healthy conception and reduces metabolic syndrome risks but also establishes a foundation for family health. These insights highlight the potential of CBA interventions to positively impact future generations.

## 1 INTRODUCTION

Creating the best possible opportunities for children is a universal value. Healthy, emotionally, and financially secure childhoods confer a lifetime of advantage.^1^, ^2^, ^3^, ^4^ In addition to vaccine‐preventable infectious diseases, many non‐communicable diseases, increasingly associated with childhood, are preventable.^5^ One such constellation of health risks includes abdominal obesity, high blood pressure, insulin resistance, hyperlipidaemia, and low high‐density lipoprotein (HDL), collectively known as metabolic syndrome (MetS).^6^ MetS can be transmitted via three pathways: lifestyle,^7^, ^8^ genetics,^9^ and epigenetics.^10^ Epigenetics is reversible,^11^ therefore, preconception adherence to healthy habits may not only reduce MetS risk in individuals,^12^ but in their offspring as well.^13^ Such a multigenerational approach to MetS prevention may be best applied to all couples irrespective of when they plan to have children and requires the involvement of both parents.

Adherence to healthy lifestyle habits is essential for reducing the risk of MetS.^7^, ^8^, ^14^, ^15^, ^16^, ^17^ However, adherence can be a challenge if socio‐ecological factors are not taken into account.^18^, ^19^, ^20^ One primary ecological factor is a person's home, the environment of which can be obesogenic.^21^, ^22^ A key social factor is an individual's partner. As a result, couples cohabitating have the potential to strengthen their chance of adherence and long‐term maintenance of a healthy lifestyle by working together. A recent systematic review of randomized control trials examined lifestyle interventions and the differences in outcomes of couples versus individual‐based lifestyle interventions.^23^ The authors concluded that targeting both members of a couple in any lifestyle intervention was more likely to have long‐lasting, positive impacts than targeting just one of the members. Additionally, spousal concordance can influence health behaviors in couples through assortative mating (i.e., choosing a spouse with similar characteristics)^24^ and cohabitation.^25^, ^26^, ^27^ Social support can improve adherence to healthy habits as couples often undertake activities such as meal preparation and grocery shopping either together or by at least involving each other in decision‐making. Consequently, a couples‐based approach (CBA) to optimizing preconception health may be an important way to reduce modifiable risks of MetS in offspring.

While the justification for a preconception CBA intervention to reduce MetS is strong, there is a paucity of such interventions reported in the literature. Therefore, this study aimed to explore the views and attitudes of each member of a nulliparous couple, who intend to start a family soon, regarding the barriers and facilitators to participation in a pre‐conception, CBA intervention.

## 2 METHODS

### 2.1 Research design and theoretical framework

This study employs Reflexive Thematic Analysis (RTA) as its qualitative research design to explore the barriers and facilitators of couples‐based lifestyle interventions. A qualitative study utilizing semi‐structured interviews with nulliparous couples was undertaken.^28^ Qualitative research is used to gain an understanding of underlying opinions, motivations, actions, and thoughts of each individual within the couples.^29^ RTA is a method for identifying, analyzing, and interpreting patterns of meaning (themes) within qualitative data. It values the researcher's subjective experience as the primary way to ascertain knowledge from data.^30^ Unlike approaches that seek to eliminate potential bias, RTA leverages the researcher's personal experiences and values as the primary instrument to make sense of the data collected. By engaging in semi‐structured, in‐depth interviews, RTA facilitates an iterative and reflective process that prioritizes the researcher's reflexivity. This approach ensures that the analysis remains flexible and responsive to the data, enabling the identification of rich, contextually grounded themes. The use of RTA in this study underscores the importance of researcher reflexivity, where the research team continuously reflects on their potential biases and influences on the research process, thereby enhancing the credibility and depth of the findings.

### 2.2 Research setting

The study was conducted online via video call in 2021 with participants from different cities in Australia. The study was conducted online due to restricted travel during the COVID‐19 lockdown in the country. At the time, all academic work was conducted online.

### 2.3 Sampling strategy, participants, and recruitment

Participants were recruited via flyers, online platforms, and by word of mouth. The included participants were couples residing in Australia, aged between 18 and 35 years and with a body mass index (BMI) ranging between 18.5 and 38 kg/m^2^. This age group is referred to as the “Millennials” having been born between mid‐1970s and 2000.^31^ In addition, participants were required to be cohabiting, in a stable relationship, and not pregnant or attempting to get pregnant for the duration of the study. Each member of the couples who agreed to participate signed a consent form and was given a unique ID to maintain anonymity. Table 1 shows the demographic information of study participants. Table 2 shows the categories and examples of questions asked to the couples.

**Table 1:** Demographic information of study participants.

Couple	Participant	Gender	Age	Relationship status
1	F1	Female	30–34	De Facto
	M1	Male	25–29	
2	F2	Female	25–29	De Facto
	M2	Male	25–29	
3	F3	Female	30–34	Married
	M3	Male	30–34	
4	F4	Female	30–34	Married
	M4	Male	30–34	
5	F5	Female	25–29	De Facto
	M5	Male	25–29	
6	F6	Female	25–29	Cohabiting (lived in the same household because of impacts from COVID‐19)
	M6	Male	30–34	
7	F7	Female	25–29	De Facto
	M7	Male	25–29	
8	F8	Female	30–34	Married
	M8	Male	30–34	
9	F9	Female	30–34	Engaged
	M9	Male	30–34	

**Table 2:** Categories and examples of questions asked to the couples.

Category	Examples of questions asked
Planning for a child	1. How long have you been thinking to have a child? 2. Have you taken any steps (are you trying to conceive right now, have you visited a GP or a fertility specialist?) 3. While you have thought about having a child, what are the reasons that have led you to wait before having a child? 4. Now lets' talk about your health. Have you thought about your own health when you think about having a child?
Previous experience of participating in a weight loss program	1. Have you ever participated in a weight management lifestyle program? a. What led you to think about your lifestyle? b. How was your experience? c. Was it a positive or negative experience? Please elaborate.
Knowledge/opinion on barriers and facilitators of weight loss programs	1. What are the most important elements of a good weight loss program? 2. How do you think we can motivate people to follow a diet and physical activity program? 3. How do you think we can encourage people to continue following the diet and physical activity even after the conclusion of the program?
Couples‐based interventions	1. What are your thoughts on involving couples in a weight management lifestyle program? a. Do you think it will be any different from a lifestyle program that targets individuals rather than couples? i. What would need to be added and why? ii. What would need to be removed and why? 2. What are your potential concerns about such a program?

### 2.4 Data collection and processing

A semi‐structured interview guide was developed as a result of discussions amongst the research team and a review of the published literature.^32^ Open‐ended questions, incorporating a nonjudgemental probing style were included to elicit in‐depth responses from those interviewed (Table 2). Interviews were conducted online using video call at a mutually agreeable time between June to October 2021. Each member of each couple was interviewed separately to ensure both voices were heard and that the answers were not led by a dominant partner. Couples were asked not to share their responses until both members of the couple had been interviewed. Each interview lasted approximately 30–60 min and was audio‐recorded using the built‐in Zoom call recorder, with a Sony recorder as a backup. The recordings were transcribed verbatim and entered into a word‐processing document for analysis by the lead author (SN). The participants were informed of the interviews being recorded during the recruitment stage and then also at the interview. Each participant was assigned a code, and transcripts were carefully reviewed to remove all identifying data to ensure participant privacy. Sampling continued until data saturation was reached, which was confirmed when no new codes emerged from subsequent interviews, leading to the cessation of sampling.

### 2.5 Data analysis

The transcripts of the recorded interviews were inputted into NVivo for analysis. A six‐phase analytical process was undertaken to analyse the interviews as recommended by Braun and Clarke^30^ including Phase One: familiarization with the data; Phase Two: generating initial codes; Phase Three: generating themes; Phase Four: reviewing potential themes; Phase Five: defining and naming theme; and Phase Six: producing the report. The author (SN) meticulously reviewed the transcripts several times to identify codes using the reflexive thematic analysis coding process.^30^ Additionally, a selective coding process using 10% of the participants was conducted by the researcher (CRKA) as a means of triangulation. Line numbers and participants' unique identifiers were used to locate the quotes. After coding, the codes were categorized under broader themes generated from the codes. Themes were finalized in consultation with all the authors of this study to ensure an accurate interpretation of the data.

### 2.6 Reflexivity

The interviewer had no prior relationship with the participants of this study. The lead researcher, conducted trial interviews before commencing the study to ensure familiarity with the interview process and to refine her interviewing skills. The research team also includes experienced researchers with extensive backgrounds in qualitative research. This team provided guidance and oversight throughout the study to ensure methodological rigor and reflexivity.

### 2.7 Ethics

Ethics approval was obtained from the University of Canberra Human Research Ethics Committee (No. 4510). informed consent was obtained from all participants.

## 3 RESULTS

Nine couples participated in the study, and since they were interviewed separately, a total of 18 interviews were collected. Participants had a self‐reported BMI between 18.5 and 38 kg/m^2^.

The couples were residing in different parts of Australia, and were from a diverse range of ethnical and cultural backgrounds. Following the sixteenth interview (eighth couple), no new or relevant information was observed, and a ninth couple was interviewed just to confirm that saturation of data had been reached.

Four distinct themes related to lifestyles and weight management emerged. The study overlapped a period of COVID‐19 pandemic lockdowns which acted as an unexpected reference point for many of the responses. In addition, each participant had attempted some form of weight management (either weight loss or weight gain) in their lives. Neither of these factors was planned or anticipated in the design of the study.

The following four themes were identified during the analysis.

### 3.1 Theme 1: Millennials are committed to preparing for their future offspring

A common desire for all couples was to be prepared for having a child. This mainly centered on career, financial security, or housing but also included formative ideas around health.

*F5: … I just really am prioritizing my career path and setting out sustainable and secure career for me to have that flexibility. A big thing for me is being financially secure throughout having children…*

*F7: we want to set ourselves up a bit more financially. So, we're looking at getting a house and we want to try and be quite deliberate with our money, and kind of set ourselves a good foundation…*

Despite not imminently considering pregnancy, some participants had already thought about the importance of reproductive health. Issues around healthy dietary intake, exercise, and psychological well‐being were raised:

*F5: …I think mental wellbeing plays a big part in my lifestyle as well. So just making sure that mentally I'm prepared for those changes should they happen. That's more so because but my mum experienced postnatal depression…*

*F4: I guess mainly, like, nutrition, alcohol, like stress as well trying to minimize stress, so that I don't… pass on stress hormones to the baby or something….*

*M8: By the time… we have a baby… whatever time it may be, I would like to be in a good fitness form to be able to play with my own kids engage with their activities…*

Women talked about the importance of entering the antenatal period healthy to be strong enough to withstand the additional pressures that come with being pregnant as the following participant stated,

F4: *I guess, like still being able to walk and do things, do housework and be physically strong without too much pain…*

It was also acknowledged that if parents are healthy there is a greater likelihood that offspring will be healthy too.

*F7: I know, both from a toll on my body perspective, but also for the health of having a baby, that it's really important to try and be as healthy as possible and look after yourself…*

For almost all participants the importance of good pre‐conception health for both sexes, and not just women, was novel information and seen as a sound justification for a CBA preconception intervention.

M5: *They always say that couples who work out together stay together. And, yeah, obviously, it's a motivation benefit. If your partner is invested in (a CBA) program and the outcome, I think you're far more likely to do it. And then I think the overall goal, like of the idea of improving your child's health, or your future child's health is also a motivational factor.*

### 3.2 Theme 2: Millennials know the features of a good program and are likely to have high adherence to long‐term behavior change

The couples reported following various healthy lifestyle programs during their lives. This enabled them to provide valuable insight regarding what an effective CBA lifestyle intervention may look like:

*M6: …just like a simple exercise routine, that's not too hard on the body, I guess. Like for example, you're coming home from work, or just before work, you don't want to burn yourself out. Like, where you can't move… afterwards. Short exercises, short bursts…*

Most male participants reported exercising at the gym to gain muscle and manage stress. A minority were engaged in such activities to address overweight. Most men had good knowledge of nutrition and spoke about regular involvement in meal preparation. They described their experience with following healthy lifestyle programs or designing their own from the internet.

*M7: …So, I think it's important not just to provide… an alternative food like program but also to people with the knowledge of why it's beneficial and what you're putting into your body… So, they are equipped with the knowledge to do it in a beneficial healthy manner not just sort of adding what, what you know what's yummy…*

*M7: …you kind of go back into other habits unless you've been educated along the way…*

*Being educated about good nutrition stimulated healthier habits for many:*

*M9: …I started to actually learn how to cook vegetables and, and um… seafood as well like fish and stuff in a way that I actually enjoyed. So, I've now kind of got a bunch of vegetarian dishes that I've added to my repertoire that I never would have dreamed of ever adding…*

*M9: Like my whole life, I didn't really enjoy dieting. I used to make a lot of jokes about vegans and vegetarians. And then all of a sudden, I'm cooking vegetarian and vegan food at home… I still eat a lot of meat but that came from me deciding you know what, I'm older. I can't keep putting shit through my body…*

Some male participants claimed to enjoy the social aspect of gym attendance and often had gym buddies. This comradery was a motivating factor for many to exercise regularly:

*M3: …Yeah, absolutely need that. Even when they start missing gym, obviously, for example, due to the cold weather that we're having at the moment, I kind of lose the motivation to go there… So, there are those things that you absolutely need to say gym buddy or like, very, you have to be very strongly motivated to do it on your own…*

Some changed their motivation to go to the gym from vanity to health,

*M4: Doing it for the right reasons… not doing it to have a certain image or whatever…*

*M4: So, I'd put healthy guidelines around what you want to achieve and, making sure it's about health and a healthy mind as well, about what you want to work towards instead of just… (how you look) …*

Female participants were more likely to have experienced weight fluctuations and disordered eating at one point or another in their lives. The consensus was that it was ideal to maintain a healthy weight, especially regarding pre‐conception health. As such, a weight education component should be integrated into any CBA intervention:

F5: *… it is something that I have had issues within the past. I've always been quite lean. And I did experience disordered eating at a younger age, where I lost quite a significant amount of weight, and had to gain weight as to become healthier…*

F8: …*I know that the only issue I have is weight. It's difficult to conceive stuff like that. Obviously, we haven't started trying. So, I can't say that it's going to be an issue for me. But every gynecologist you go to the obvious say that you shouldn't have any belly fat for your ovaries and for your uterus to work properly.*

### 3.3 Theme 3: Millennials acknowledge the importance of CBA programs to support healthy lifestyles

There was enthusiastic support for the idea of a CBA healthy lifestyle program, particularly from women. Men appeared generally more reticent because they feared having to cease existing gym routines, but then some saw such programs as motivation to work together.

*M8: One of the things I feel is, engagement of a second person, what I've observed all the time, is people who do these programs in pairs, they tend to become consistent for a long time… sometimes motivate each other, because if person A is achieving 100% of the targets and person B's achieving 85%, they'll motivate Person B to achieve 15% more next time.*

Couples generally found it easier to adhere to healthier habits because of their partner's support. Participants felt that CBA healthy lifestyle programs should ideally encourage joint menu planning and food purchasing.

*M7: Usually, it's sort of the partner and I will put together like a grocery list, say on like, a Saturday or a Sunday, and we'll go through the meals that we want to cook and it's usually quite healthy, we find that if we don't do our weekly shop, then we don't have meals through the week, then we can't be bothered a lot of the time to do shopping through the week after work… And it's relatively healthy stuff*.

*F2: Well, I can tell you from experience, like he kept me going, because, when I come from work and I think of like, we're going to have that boring chicken again for dinner like, he'll just cook it ahead and, make a salad with…*

Some participants found it easier to incorporate more vegetables and fruit in their diet because of their partner,

*M7: Yes, like I would say I don't put a huge amount of thought into it. But my partner is a little bit more conscious of like the fruits and vegetables and she's I don't know I'm quite good at cooking, say like the protein portions of the meals like, your steak, salmon all that sort of stuff. However, she's definitely the vegetable chef in the house.*

Alternatively, other participants reported that one‐half of their partnership focused on diet and the other on physical activity. They found this situation helpful as one would push the other to exercise and the other would organize meal planning.

Social situations, especially during holidays can make weight management challenging. Strategies to cope with a ‘temporary’ change in lifestyle should be incorporated into any CBA program.

*M1: … the reason I stopped (healthy diet) was coming into Christmas. I tend to go away over Christmas and… a lot of the time when we're having a few beers and if somebody's cooked a meal, it's hard for me to say no because I'm on a diet…*.

Some participants noted that they had made several weight management attempts throughout their lives but found it difficult to adhere as they were doing it by themselves. Participants emphasized the importance of peer support to achieve desired outcomes.

*M1: …I'm somebody who would go to the extremes of crash dieting and when it wasn't working, I get upset and I would binge eat and blow out the other way…*

Any CBA healthy lifestyle program should ideally include evidence‐based nutrition advice to ensure long‐term success and adherence:

*F8: … Results‐wise, I think that Keto was the best one because I immediately lost 10 kgs in 1 month. But the worst thing about that is that the moment I will, I would stop it, I would gain double the amount of weight I lost. The other thing I did was portion control… it was very gradual. But I think it took me like a good amount of time to gain that weight back. That was a good I think that's what I want to do right now*.

### 3.4 Theme 4: Millennials appreciate that future global emergencies may require lifestyle modifications

An unanticipated finding of the interviews was the way each couple coped with the recent global pandemic. In Australia, COVID‐19 lockdowns created numerous lifestyle‐related challenges with many participants expressing that they were uninspired during this time to eat healthy and exercise regularly.

*F9: …before the lockdown, I will say that it was better because I was working in the office. I was like… how you normally snack like apple, banana. I was moving more because I had to take the train or whatever. But now because of the lockdown, it's like, you eat what you can get, maybe you eat because you are bored…*

Many participants reported experiencing:

*M6: …COVID kilos. You gain extra weight…*

For many participants such lessons emphasized the importance of CBA:

F5: *…and during this lockdown, because we're only able to exercise outside for an hour, we seem to be spending that time together instead of doing our own things (physical activity) …*

## 4 DISCUSSION

The study hypothesized that couples living together believe that CBA in a lifestyle intervention program strengthens adherence to diet and physical activity, fostering long‐term commitment to healthy habits. This is important for the reduction of multigenerational risk of MetS. While the couples in our study were not following a prescribed lifestyle program designed specifically to prevent MetS, they were preparing for the wellbeing of their future children in other ways.

Participants showed a commitment to preparing for the well‐being of their future offspring. Despite not intending to start a family imminently participants had already considered issues such as the health of each member of the couple during pregnancy and beyond in addition to financial security, and property ownership. This trend is evident in most developed nations: to invest more in fewer offspring.^33^ The positive impacts of substantial investment in children, as supported by the social determinants of health, are well‐documented, encompassing education attainment, nutrition, physical activity, and emotional well‐being.^34^ A 2017 Dutch cross‐sectional study assessed whether actively preparing for pregnancy by women (*n* = 283) is associated with lifestyle changes during the preconception period. The authors found that almost 60% of women acquired preconception information themselves and 25% consulted a healthcare professional during this period. The former group was significantly more likely to quit drinking (adjusted odds ratio [OR] 5.46 (95% confidence interval [CI] 1.76–16.96)), improve their nutrition (adjusted OR 7.84 (95% CI 3.03–20.30)) and use folic acid (adjusted OR 3.90 (95% CI 2.00–7.62)) compared with women who did not prepare for pregnancy. Similarly, an Italian qualitative study explored the attitudes and behaviors of childbearing‐aged women regarding preconception fitness. Focus group findings indicated the presence of many barriers, a lack of awareness of preconception health by women and poor efforts in its promotion at the population level. Another Dutch study of 229 men explored their perceptions regarding the need to engage in pre‐conception care.^35^ Most men did not access preconception information (59.0%) nor visited a preconception consult (79.5%). Men who categorized their preconception lifestyle as unhealthy (score 6 out of 10) less often retrieved information (adjusted OR 0.36 [95% CI 0.14–0.93]) than men with a healthy preconception lifestyle. While several men expressed their fear of infertility, this did not lead to an increased uptake of preconception care as men felt they were healthy enough already. The authors concluded that despite high awareness of the positive influence of a healthy lifestyle, men's perceived need for preparing for pregnancy remains low. Similarly, the findings of our study indicate that although couples were not aware of the epigenetic transmission of MetS, they did have a plan for maintaining their health for fertility and pregnancy.

Poor health outcomes in adults can be traced back to the quality of parental nutrition at the time of conception as evidenced by the seminal Dutch^35^, ^36^ and Chinese famine studies.^10^ Tailoring preconception information towards the specific needs of both males and females has the potential to provide a window of opportunity to improve both members of a couple's reproductive health and possibly the health of future generations.

Designing a successful intervention requires knowledge of what has worked or did not work in the past for the target population. In our study, all the couples interviewed had, at some point, engaged in either diet, physical activity, or both with the explicit intention of improving health. While this partly aligns with national trends where nearly a quarter of adults aged 18 and above meet physical activity guidelines^37^, ^38^ current dietary practices however present a concerning pattern. According to the Australian Institute of Health and Welfare (AIHW), there is a high intake of nutritionally poor and energy‐dense foods, coupled with insufficient consumption of the recommended five food groups—vegetables, fruit, grains, meat, and alternatives, and dairy products and alternatives.^39^ This disjunction between recommended dietary practices and actual consumption shapes the backdrop against which our participants bring valuable experiences to critique the concept of lifestyle interventions for couples. Their diverse encounters with diet and physical activity provide a rich foundation for understanding the complexities and nuances that any intervention must navigate to be effective.

Participants in our study overwhelmingly expressed support for involving their partners in healthy lifestyle programs, highlighting the crucial roles of support, goal setting, knowledge, and motivation. They emphasized the significance of empowering individuals with knowledge about nutrition, physical activity, and the overall benefits of a healthy lifestyle. Supporting our findings, a 2014 Croatian randomized single‐blind study (*n* = 124) investigated predictors of attrition and successful weight loss in the management of obesity. Marital status and outpatient education were the strongest predictors of 12‐month weight loss, underscoring the influence of interpersonal dynamics and educational background in achieving positive health outcomes.^40^ Similarly, a 2013 cross‐sectional study (*n* = 70 couples) explored the impact of spousal support on diabetes control through exercise. The findings revealed that spousal support significantly increased exercise adherence, contributing to improved diabetes management in individuals with diabetes.^41^ These studies provide empirical evidence reinforcing the participant‐voiced importance of partner involvement and support in health‐related interventions.

Partners or spouses living together form the core of the socio‐ecological model of health^18^, ^20^ by cohabiting the shared space and by having similar lifestyles. This proximity and shared environment lay the foundation for the interconnected health dynamics within a couple. Post‐intervention adherence has been a notable outcome in CBA studies, reflecting the influence of mutual support and shared health goals. In a randomized controlled trial by Trief et al.,^42^ targeting couples where one member had diabetes, the role of spousal support in diabetic control and weight management was investigated. The study revealed that individuals with chronic illnesses, such as diabetes, can significantly benefit from the support of their partner in interventions, leading to long‐term positive outcomes. CBA interventions have also demonstrated effectiveness in diverse health contexts, including HIV,^43^ cancer^44^, ^45^ and dietary habits.^46^ However, it's worth noting that existing examples of CBA interventions often involve one member as the primary patient, with the partner participating primarily for support.^23^ A true CBA intervention, as acknowledged by participants in our study, would go beyond traditional roles, leveraging each member's strengths while providing mutual support for any shortcomings for instance, if one participant excelled in incorporating vegetables into their diet, their partner might be adept at integrating exercise into their daily routine. This collaborative approach, where both partners actively contribute to the intervention based on their strengths, reflects a more holistic and synergistic model of CBA health promotion.

In addition to the dynamics within the couple, external factors, including the influence of social networks and individual preferences, were reported by participants in our study. Some male participants, for instance, found motivation in having a gym buddy outside the couple, recognizing that their preferred exercises differed from those of their partners.^47^ Maintaining an active social life not only enriches a couple's connection to each other but others outside their relationship as well in addition to serving as a protective factor against depression in later life.^47^, ^48^ Interestingly, while there was less enthusiasm among male participants for having their partners as gym buddies, they were very positive about collaborating with their partner regarding the dietary aspect of any CBA intervention. One such aspect which arose in our study is advanced meal preparation which aligns with the findings of the 2017 NutriNet‐Santé study.^49^ This web‐based research involved 40,554 participants and demonstrated that strategic meal planning is associated with a healthier diet leading to a reduced risk of obesity. These insights underscore the positive impact of intentional meal preparation on overall well‐being, emphasizing the multifaceted nature of health interventions within couples.^49^

The unforeseen alignment of our study with the COVID‐19 lockdowns offered a poignant moment for reflection among participants. During the pandemic, spousal concordance became evident through observed changes in the dietary and physical activity habits of couples. Participants reported an increase in unhealthy food consumption patterns, mirroring global trends during the challenging circumstances. Intriguingly, amidst these dietary changes, there was also a reported increase in physical activity, with couples engaging in activities such as long walks together during the allotted “time allowed outside."^50^ These findings resonate with a UK‐based longitudinal study involving 264 participants, where challenges in regulating eating habits during the COVID‐19 lockdown were reported. However, similarly to our observations, participants in the UK study indicated an increase in exercise during this period, highlighting a dual impact on lifestyle behaviors.^51^ The unexpected overlap with the pandemic in our study not only offers a unique perspective on couples' health behaviors but also aligns with broader patterns observed globally during times of heightened stress and restricted movement.^51^

While not the primary focus of this study, concerns about fertility impacts of COVID and of COVID prevention (specifically vaccination) were voiced, highlighting the pervasive confusion amidst the influx of information during this uncertain time. According to the ABS, fertility rates dropped to an all‐time low of 1.58 births per woman in 2020 in Australia.^52^ The Families in Australia Survey reported that of the 12% of respondents who had been trying to conceive before the pandemic, 18% stopped trying as a direct or indirect result of the pandemic.^53^ The pandemic illuminated the importance to potential parents of creating optimal opportunities for a good and healthy life for each child born. This heightened emphasis on reproductive health amid global challenges serves as a powerful motivation to redirect attention to couples, empowering them with the knowledge to address MetS and the ongoing global obesity epidemic.^54^

## 5 STRENGTHS AND LIMITATIONS

The study's demographic focus on 25–35‐year‐olds living in Australia limits the generalizability of findings to other countries or all potential parents. However, a notable strength lies in the multiple ethnicities of selected participants, a feature of modern, multicultural Australia. Conducted during the COVID‐19 pandemic, the study's timeline incorporates major disruptions and a period that fostered reflection on values, adding a unique contextual layer. A limitation of the timeline is that it captures a period marked by significant disruptions rather than a representation of a nondisruptive lifestyle. Furthermore, as none of the couples had prior experience with a CBA lifestyle program, their opinions were based on personal opinions rather than actual experiences in such programs aimed to maximize pre‐conception health. However, each member of the couple brought personal experiences of weight management through diet, physical activity, or both, thus providing a wealth of lived experiences. While these limitations need consideration, they underscore the need for future research to explore couples' responses in varied contexts and program experiences.

## 6 IMPLICATIONS FOR PRACTICE, POLICY, AND FUTURE RESEARCH

The insights gained from couples' perspectives on joint lifestyle behaviors, including healthy diet and physical activity, as investigated in this study, advocate for the implementation of true CBA lifestyle programs in future research. Empowering couples to address preconception poor lifestyle choices, alongside harmful substance consumption, holds the potential to create optimal outcomes for the next generation. This approach may play a crucial role in mitigating the global pandemic of obesity and related health issues, including MetS. The study underscores the importance of integrating couples into interventions aimed at fostering healthier lifestyles, thereby contributing to broader public health goals. Moving forward, research should focus on implementing and evaluating such CBA lifestyle programs to assess their effectiveness and impact on long‐term health outcomes for couples and their offspring.

## 7 CONCLUSION

This study revealed a notable level of intention among millennial couples interviewed to create the best emotional, financial, and health circumstances for their offspring. These couples expressed support for the concept of CBA lifestyle interventions as a proactive measure to address potential epigenetic risks to their children. Importantly, the study found that couples believed having their partner in the same program would enhance adherence to the diet and physical activity plan, facilitating the long‐term maintenance of healthy habits. The findings strongly advocate for the exploration and testing of CBA interventions, ensuring equal targeting of both members of a couple. Adherence to healthy habits, as promoted by such interventions, not only contributes to healthy conception and reduces the risk of transmitting metabolic syndrome but also sets a new family on a trajectory of health from the outset. The study participants emphasized the importance of couples participating in lifestyle programs together to safeguard their well‐being and the future health of their offspring. These insights underscore the potential of tailored CBA interventions to positively impact family health and lay the foundation for healthier generations.

## AUTHOR CONTRIBUTIONS

**Sundus Nizamani**: Writing—original draft; conceptualization; investigation; methodology; validation; data curation; formal analysis; software. **Catherine R. Knight Agarwal**: Writing—review and editing; methodology; supervision; data curation. **Shawn Somerset**: Conceptualization; writing—review and editing; methodology; supervision. **R. A. McFarlane**: Supervised; methodology; formal analysis; investigation; resources; data curation; writing—review and editing. All authors have read and approved the final version of the manuscript, had full access to all of the data in this study, and take complete responsibility for the integrity of the data and the accuracy of the data analysis.

## CONFLICT OF INTEREST STATEMENT

The authors declare no conflicts of interest.

## TRANSPARENCY STATEMENT

The lead author Sundus Nizamani affirms that this manuscript is an honest, accurate, and transparent account of the study being reported; that no important aspects of the study have been omitted; and that any discrepancies from the study as planned (and, if relevant, registered) have been explained.

## Data Availability

The data that support the findings of this study are available from the corresponding author upon reasonable request. The data that support the findings of this study are available on request from the corresponding author.
